# Toxicology of Nanomaterials: Permanent interactive learning

**DOI:** 10.1186/1743-8977-6-28

**Published:** 2009-10-28

**Authors:** Paul Borm, Vince Castranova

**Affiliations:** 1 Zuyd University, Centre of Expertise in Life Sciences, PO Box 550, 6400 AN, Heerlen, The Netherlands; 2 Pathology and Physiology Research Branch, NIOSH Health Effects Laboratory Division Morgantown, West-Virginia, USA

## Abstract

Particle and Fibre Toxicology wants to play a decisive role in a time where particle research is challenged and driven by the developments and applications of nanomaterials. This aim is not merely quantitative in publishing a given number of papers on nanomaterials, but also qualitatively since the field of nanotoxicology is rapidly emerging and benchmarks for good science are needed. Since then a number of things have happened that merit further analysis. The interactive learning issue is best shown by report and communications on the toxicology of multi-wall carbon nanotubes (CNT). A special workshop on the CNT has now been organized twice in Nagano (Japan) and this editorial contains a summary of the most important outcomes. Finally, we take the opportunity discuss some recent reports from the nanotech literature, and more specifically a Chinese study that claims severe consequences of nanoparticle exposure.

## Carbon Nanotubes

A recent series of publications within the past year on carbon nanotubes (CNT) have created extensive interest among regulatory agencies internationally. The first paper by Takagi et al. [1] reported that intraperitoneal injection of MWCNT resulted in mesothelioma in p53 +/- mice. Issues raised concerning this study were the high dose (3 mg/mouse) used and the relevance of the p53 +/- mouse model. Soon after, Poland et al. [2] reported that abdominal injection of long MWCNT but not short MWCNT induced inflammation and granulomatous lesions on the abdominal side of the diaphragm at one week post-exposure. In contrast to the Takagi et al. [1] study, they used wild type mice and a much lower dose (50 μg/mouse) of MWCNT. Although this study documented acute inflammation, it did not evaluate whether this inflammation would persist and progress to mesothelioma. Sakamoto et al. [3] used a 240 μg/rat dose of MWCNT injected intrascrotally and reported mesothelioma of the abdominal and thoracic lining 40 weeks post-exposure. Although this was a much lower dose than used by Takagi et al. [1], some have expressed concern about the dose rate employed using a single injection. Recently, Muller et al. [4] reported that no mesothelioma was noted 2 years after intraperitoneal injection of MWCNT (2-20 mg/rat). However, the MWCNT sample used in this study was very short (< 1 μm), and the study by Poland et al [2] would have predicted such short nanotubes to express low activity. An issue that still remains is whether inhaled MWCNT would reach the intrapleual space. Preliminary results from Hubbs et al. [5] indicate the such migration can occur in that they report microscopic evidence that MWCNT had penetrated the pleural surface of the lung 56 days after pharyngeal aspiration of 40 μg of MWCNT in a mouse model.

In light of these reports of asbestos - like properties of CNT, a panel discussion was held to evaluate the state of CNT toxicology at the International Nanofiber Symposium - 2009 in Tokyo, Japan on June 19, 2009. This discussion was followed by a Nanofiber Extended Workshop on CNT Toxicology and Safety in Nagano, Japan on June 22, 2009 organized by Dr. Morinobu Endo from Shinshu University, Nagano, Japan and Dr. Shuji Tsuruoka from Mitsui & Company, Ltd., Tokyo, Japan.

The brainstorming panel discussion group at the Nanofiber Symposium - 2009 included the following international experts on carbon nanotubes and nanotoxicology:

1. Dr. Gunter Oberdorster, University of Rochester, USA

2. Dr. Nicklas R. Jacobsen, National Research Centre for the Working Environment, Denmark

3. Dr. Roel Schins, Institut fur Umweltmedizinische Forschung, Germany

4. Dr. Vincent Castranova, National Institute for Occupational Safety and Health, USA

5. Dr. Morinobu Endo, Shinshu University, Japan.

In light of the recent intraperitoneal studies with MWCNT, the panel discussion centered on the similarities and differences between CNT and asbestos. CNT are long and thin fibrous structures like asbestos. The panel noted the importance of the 3 D's in fiber pathogenicity, that is, dose, dimension, and durability. Thus far, doses used in intraperitoneal instillation studies with MWCNT have been high, and dose rate has been immediate rather than over months or years. Critical issues include: the need for data concerning occupational exposure to CNT and information concerning the rate in which inhaled CNT can migrate to the intrapleural space. Poland et al. [2] noted the unique bioactivity of long (20 μm) CNT. Such long fibers would cause "frustrated phagocytosis", which has been reported to result in prolonged release of oxidants and inflammatory mediators from alveolar macrophages, leading to a pathological response. A knowledge gap is information concerning the expected length distribution of CNT aerosolized in workplace environments. Data to date suggest that most CNT structures would be < 10 um in length and be completely engulfed by macrophages. CNT are often treated with acid or high temperature to remove metal catalysts, so are durable. The few long term animal studies available do not indicate that CNT dissolve or rapidly clear from deep the lung. Therefore, CNT are likely to be biopersistent. More data are needed concerning the rate of dissolution, clearance, and translocation of CNT in the lung. In addition to the activity of CNT following intraperitoneal injection, aspiration or inhalation of CNT has been reported to cause persistent fibrosis [6]. In light of this bioactivity, the panel stated that it would be prudent to control exposure to CNT through containment, ventilation, and use of personal protective equipment.

## Workshop Carbon Nanotubes- Nagano

At the Nanofiber Extended Workshop on CNT Toxicology and Safety in Nagano, Japan, a group of international scientists presented current information concerning the applications as well as implications of CNT. Dr. Aoki presented data indicating that MWCNT were biocompatible within an inplant, caused formation of new bone, and inhibited osteoclasts through NFAT. Dr. Akiba described the incorporation of CNT into fibers for use in making anti-static fabric, non-metal wire sensors, and heating fabric for water tanks, car seats and other applications. Dr. Oberdorster discussed several issues to consider in conducting nanotoxicological research, such as, dose rate, modification of the activity of the CNT surface by adsorption of protein and/or lipid from biological fluids, functionalization of the CNT surface to decrease toxicity, and the need for studies to evaluate the distribution and biokinetics of CNT after pulmonary exposure. Dr. Schins presented in vitro responses to CNT exposure. He emphasized the role of in vitro studies to elucidate mechanisms involved in pulmonary responses. Dr. Jacobsen also addressed the relevance of in vitro results to in vivo responses. He noted the issue of the use of doses in vitro which are relevant to in vivo doses, i.e., ug/cell surface. He reported that in vitro CNT can adsorb nutrients from the medium to exhibit artifactual inhibitory effects on cell proliferation at high CNT/cell surface doses. He also discussed methods used in his institute to determine the "dustiness" or ease of aerosolization of CNT under workplace-like conditions. Dr. Kanno presented a new project which extended their original intraperitoneal instillation studies with MWCNT. These studies used lower doses then the original published investigation and still found mesothelioma. Dr. Castranova reported pulmonary inflammatory and fibrotic responses to aspiration of MWCNT. He also presented a progress report of an ongoing inhalation study of MWCNT showing acute responses similar to those found after pharyngeal aspiration. In conclusion, the scientific panel identified several goals for future research: the need to have well characterized standard test materials, the need to harmonize assay systems, the need to use relevant in vitro and in vivo doses, and the need to understand occupational exposure levels and workplace processes associated with exposure. Such information is vital for risk assessment. Until these data are available, exposure control is prudent.

Apart from carbon nanotubes there are many interesting debates in the field such as that about the best metrics for measuring and judging nanoparticle properties. Recent input from the nano-community was given in a review article by Auffan et al [7]. The authors reviewed the size-dependent properties of a variety of inorganic nanoparticles, mostly metal oxide nanoparticles, and found that particles larger than about 30 nm do not in general show properties that would require extra regulatory focus. The interesting thing about this paper is that it is based on physicochemical particle properties such as melting point and Curie-temperature rather than bioactivity. In summary, it takes the particle chemical reactivity as a starting point, and comes to a surprising recommendation which is worth considering. However, the authors are either unaware or simply not using the fact that biological response is critically important. A summation of the importance of internal dose and particle surface area to bioactivity has recently been reviewed [8]. Nevertheless, the generation of such an inverse hypothesis into toxicology is useful to an appropriate understanding of issues facing risk assessment for nanoparticles. This is a good example of interactive learning in the scientific community.

## Fatal workplace exposure?

Despite all these creative, interactive efforts that are gradually building concerning the conceptual understanding of nanoparticle toxicology, one must remain very critical in assessing nanotoxicology studies. Increasingly, papers describing new effects of existing particles or new nanomaterials are being published. Often issues of concentration and exposure conditions are not put into the perspective of real-life use of nanomaterials. The number of papers elucidating hazards of nanoparticles is rapidly increasing, but one must be cautious in making broad conclusions. A case in point is a recent paper by Song, Li and Dy [9]. In their paper, the authors describe the clinical course of 7 young female workers (aged 17-47 years) that were exposed to a polyacrylate spray which contained a cocktail of 6 or more organic chemicals as well as presumably nanoparticles. Spray painting of this coating in an unventilated room with no personal protection led to hospital admissions within 13 months of exposure, associated with shortness of breath and pleural effusions. All workers had extremely low lung function values, elevated lymphocytes and neutrophils in lavage, widened septa in the alveolar region, and signs of pulmonary fibrosis. Two of the workers died as a result of exposure. Although particles were identified in lung biopsies and lung fluids, no chemical identification was conducted to determine whether these particles were of environmental (diesel or cooking smoke particles) or occupational origin. In addition, the presence of nanoparticles in workplace air was not determined. This adds to the uncertainty of the conclusion that workplace nanoparticles were the causative agent. An additional error the authors made was the generalization of their findings to all nanoparticles, both in the title and abstract of the paper. Therefore, evidence supporting such a strong title remains to be gathered. In the meantime, we have new opportunities for debates and interactive learning.
